# Effect of Heat Therapy on Pain During the First Stage of Labor Among Primigravid Women: A Pilot Study

**DOI:** 10.7759/cureus.77517

**Published:** 2025-01-15

**Authors:** Isabel Lawot, Imran Khan, Nitika Thakur, Tumla Shrestha

**Affiliations:** 1 Department of Nursing, Sharda School of Nursing Science and Research, Sharda University, Greater Noida, IND; 2 Department of Midwifery, Maharajgunj Nursing Campus, Institute of Medicine, Tribhuvan University, Kathmandu, NPL; 3 Department of Medical-Surgical Nursing, Sharda School of Nursing Science and Research, Sharda University, Greater Noida, IND; 4 Department of Pediatrics, Sharda School of Nursing Science and Research, Sharda University, Greater Noida, IND; 5 Department of Child Health Nursing, Maharajgunj Nursing Campus, Institute of Medicine, Tribhuvan University, Kathmandu, NPL

**Keywords:** heat, labor pain, primigravida, therapy, women

## Abstract

Background

Labor pain is often one of the most severe pains a woman may feel in her lifetime, and childbirth is usually considered one of the most physically demanding experiences a woman may have. An effective and safe midwifery intervention for reducing labor pain in pregnant women is the administration of heat.

Aim

The objective of the article is to compare the effectiveness of lumbosacral heat application on pain throughout the active first stage of labor between the interventional and control groups.

Method

A randomized controlled trial among 10 low-risk primigravid women admitted to Bharatpur Hospital in Chitwan, Nepal, was carried out to evaluate the intensity of pain and duration of the active first phase of labor by allocating them randomly into intervention (5) and control groups (5); one group received heat therapy on the lumbosacral region, while the other received standard care. The Visual Analog Scale (VAS) was applied as an instrument to assess pain four times: before heat therapy at 4-5 cm of cervix dilation and three times after heat application at 4-5, 7-8, and 9-10 cm. The data were analyzed descriptively and inferentially using a repeated measures ANOVA test.

Results

The mean age of the mothers was 21.80±3.327, most of them belonged to the low socioeconomic class, all of them were living in joint families, 80% of participants had less than 18.5 body mass index, all participants had visited an antenatal clinic for check-ups, and 60% had spontaneous labor started. The findings show a higher labor pain score before intervention (mean=6.80) which progressively decreased after an intervention: post-test 2 (M=6.20; SD=0.83), post-test 3 (M=5.60; SD=0.89), and post-test 4 (M=5.00; SD=0.70). The mean, standard deviation, and F-value of labor pain before and after the application of heat therapy among primigravid women indicated a significant mean difference in labor pain (F (3,12)=4.500; MES=0.66; p=0.025; η2=0.53) with a large effect size in contrast to the control group. The p-value for post-test 4 was 0.025, representing that the null hypothesis was rejected. This acclaims that heat therapy has an effect on labor pain.

Conclusion

It is concluded that the application of heat on the lower back during labor lowered the pain level experienced by primigravid women in the interventional group compared to those in the control group. Thus, heat therapy can be considered a viable method for managing labor pain.

## Introduction

Childbirth is one of the most significant and complex experiences in any mother's life [1]. Horsch and Ayers stated that childbirth is often described as one of the most physically demanding experiences a woman can go through. The prenatal period is a complicated physiological process encompassing numerous identical processes involved in stress responses. About 15-20% of mothers in Western countries describe their childbearing as traumatic which could be prevented [2]. Aziato et al. stated that women's experiences with labor often lead them to expect pain with distressing anxiety, panic, and depression, especially when the labor process is prolonged [3].

The study by Nori et al. described that managing labor pain is a tough situation for both healthcare workers and expectant mothers [4]. While epidural analgesic pain relief is considered the benchmark, it carries health hazards for both the parturient and newborn. Labor and Maguire compared the pattern of labor pain between nulliparous and multiparous women, with well-documented evidence showing that pain scores are generally higher in nulliparous women compared to multiparous women [5]. Again, Aziato et al. reported that labor pain ranged from mild to intense and is experienced all over the body, especially in the lower belly, in the vagina, and around the waist. The women cried, screamed, and shouted to communicate their childbirth distress [3].

A study by Akadri and Odelola reported that the average pain experience of parturients, as assessed using the Visual Analog Scale (VAS) instrument, was 7 with a range of 1-10. Fifty percent of parturients estimated labor pain to be as severe as 7.1. The participants (86.4%) wished for available pain management. Participants with a more-than-normal body mass index (BMI), more-than-normal birth weight of their babies, higher educational status, and gestational age of more than 37 weeks had statistically significant associations with pain experience [6]. Similarly, a study in Nepal published that 32% of parturients rated their labor pain as severe, 57% moderate, and 11% mild. Comparing the labor pain experience between the high-risk age group of less than 19 and more than 35 with the normal childbearing age group of 20-34 years of age, 30% of the high-risk pregnant women group reported having severe labor pain. Only 20.7% of nulliparous parturients classified it as severe, compared to those pregnant for the second time (37%) [7].

In this study, Gregolis et al. stated the length of labor is another crucial factor for mothers, as it takes part in their pain experience through childbirth. A systematic review disclosed that the duration of the active first stage of labor among interventions of hot or warm showers was less than in the control group (221.2 versus 312.6 minutes) [8].

Despite improvement in pain relief options, Beyable et al. have shown concern about labor pain which was frequently disregarded especially in developing countries [9]. In another study, the authors have shown concern about using pharmacological methods that can result in several dangerous adverse effects for both mother and child, including reduced cardiac output, nerve impairment, and allergies, and can prolong the second stage of labor. Many healthcare workers expressed their experiences in employing non-pharmacological approaches when attending to women through the labor process to reduce pain, and they highlighted that these measures are safer for the mother and the baby [10].

Consequently, the study performed by Cavalcanti et al. encouraged the application of complementary therapies as an alternative method of pain management throughout the first stage of labor which is affiliated with national and international strategies for pregnancy and childbirth care. Also, this study hypothesized that there is no statistical difference in the level of pain during the active first stage of labor among primigravid women [11].

## Materials and methods

Research design

This study is an experimental randomized controlled trial that used a posttest-only control group design to assess the effect of heat therapy on pain throughout the active first stage of labor among primigravid women who did not have any active complications during pregnancy. Random allocation was used to split the participants into two groups: the intervention and control groups.

Ethical consideration

Written approval was obtained from the authority of Sharda School of Nursing Science and Research, Sharda University, Greater Noida, to carry out the study. Ethical clearance was approved by the Ethical Review Board of Nepal Health Research Council (NHRC) (approval number: 945) and the relevant authority in Bharatpur Hospital in Chitwan, Nepal, where the trial was conducted. Informed written agreement was also gotten from the participants. Data for the pilot study was collected between February 25 and March 7, 2024. The study comes under the trial registered on ClinicalTrials.gov with the ID NCT06214585.

Participants

This study recruited primigravid women, aged 18-35 years, with low-risk pregnancies admitted at the maternity ward of Bharatpur Hospital. Eligible participants anticipated a normal delivery with a fetus of normal size weighing 2500-3500 g as confirmed by an ultrasound scan within 37 and 41 weeks of gestation. Involved participants were those who had not used analgesics throughout the first stage of labor. The study included women in the early stages of labor. The pilot study was carried out at Bharatpur Hospital in Chitwan, Nepal.

Intervention

In total, 10 participants were recruited for this pilot study who were admitted to Bharatpur Hospital and anticipated to have a normal vaginal delivery. The eligible primigravid women were selected purposively for the study. After recording the demographic and obstetric data, the participants were enrolled randomly into the intervention and control groups using opaque envelopes. To prevent contamination, the intervention and control groups were kept separate. To ensure a better understanding, the researcher explained all the procedures of the heat therapy protocol again. For the intervention group, a rubber hot water bag with a capacity of two liters was used to apply on the lumbosacral region of the participants throughout the active first stage of labor including all the maternity care provided during labor according to hospital policy. The hot water inside the bag was kept at a temperature of 40-42°C. The heat was applied hourly for 20 minutes until full cervical dilation. The tap water was heated up to 70°C and checked with a laboratory thermometer before filling the bag. The hot water bag was covered with a towel and placed on the lumbosacral region of the parturient, making sure that the heat was comfortable for them. After 10 minutes of application, the water was replaced when the temperature dropped below the appropriate level.

Once the cervix had dilated by around 4-5 cm following a vaginal examination by a healthcare provider and it was documented in a partograph, heat therapy was started for primigravid women in the active first phase of labor. Before the application of heat therapy, the intensity of pain was estimated with the VAS. After the intervention, the pain was assessed at 4-5, 7-8, and 9-10 cm of cervical dilation for both groups. The control group received all the regular maternity care provided by the hospital as per their policy except heat therapy.

Outcome measures

Pain levels were reassessed immediately after the first heat application at 4-5 cm, 7-8 cm, and 9-10 cm dilation of the cervix as measured using vaginal examination by a healthcare provider and documented in a partograph. The VAS was the instrument used to evaluate the outcome of the intervention. It was a pain grading scale first used by Hayes and Patterson in 1921. The self-reported action of symptoms are used to compute scores, and each handwritten mark is positioned at a single point along a 10 cm line that signifies a range between the two ends of the scale: "no pain" is at the left end (0) and "worst pain" is at the right end (10) [12].

Sample size of the study

The sample size for the main study was estimated using the G*Power statistical software (Ver. 3.1.9.4, Heinrich-Heine-Universität Düsseldorf, Düsseldorf, Germany), with a significance level of 0.05. According to the software, a sample size of 53 participants per group was required to achieve an 80% power (with a 20% error margin). Therefore, for this pilot study, 10% of the sample was included, that is, five samples in each group.

Randomization

Primigravid women admitted to the maternity ward of Bharatpur Hospital for spontaneous delivery were selected through purposive sampling. Participants were randomly assigned into two groups using opaque envelopes. Since blinding was not feasible for participants and care providers, only the outcome was blinded.

Data analysis procedures

The data was arranged and input into SPSS for Windows, Version 16.0 (Released 2007; SPSS Inc., Chicago, Illinois, United States) for analysis. Descriptive and inferential statistics were applied to analyze the information in alignment with the study objectives. The demographic information was calculated using frequency and percent, and the relationship was calculated using inferential statistics such as the repeated measures ANOVA test.

## Results

**Figure 1:**
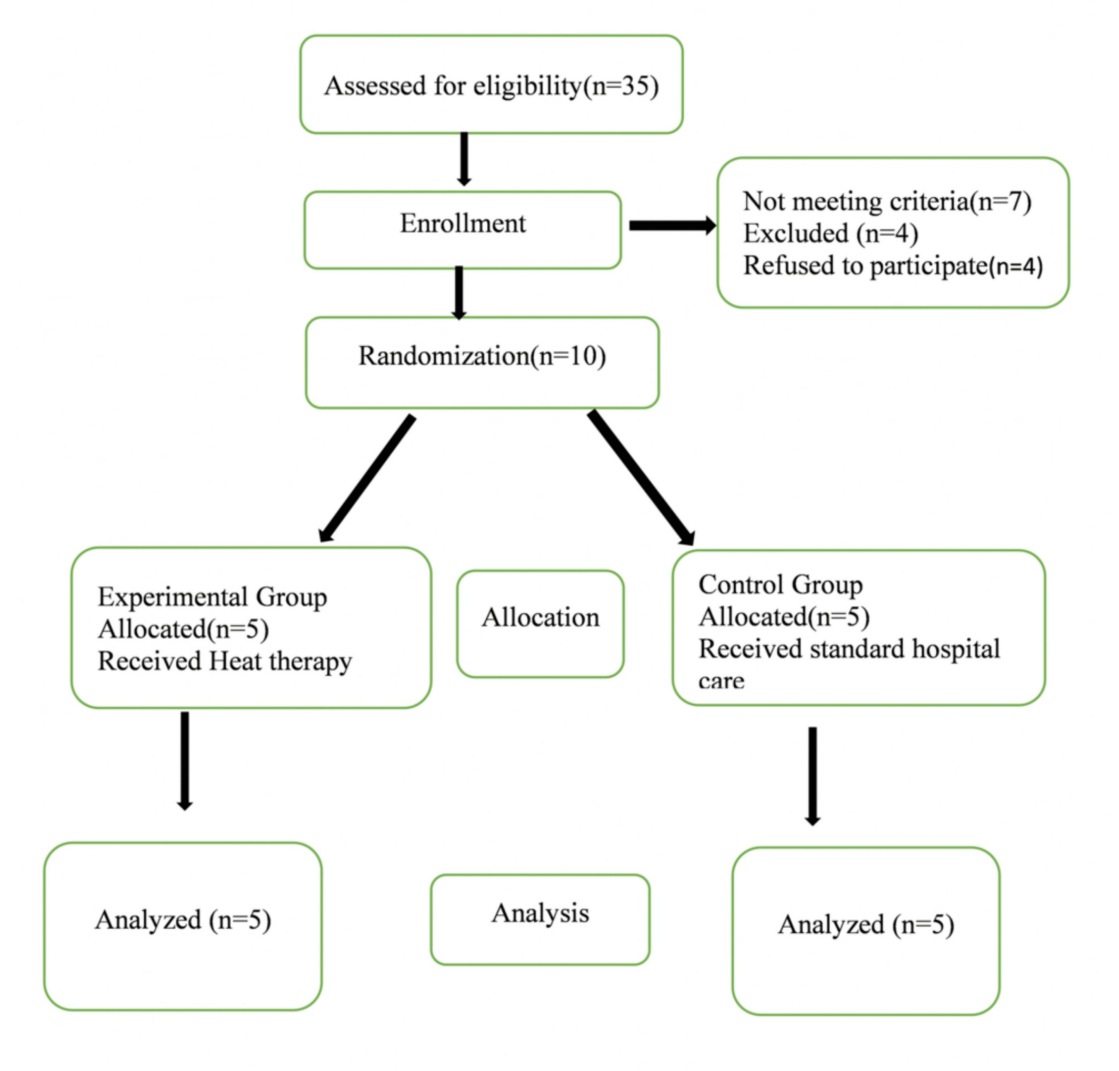
CONSORT flowchart of assessment, enrollment, and analysis CONSORT: Consolidated Standards of Reporting Trials

A total of 35 participants were screened for eligibility after obtaining informed consent. Seven were considered ineligible, four failed to participate, and four were excluded due to developing complications. Ultimately, 10 participants were enlisted and separated into two groups. One group received heat therapy as an intervention along with standard care, whereas the control group was provided standard care (maternal healthcare) according to the hospital policy. A Consolidated Standards of Reporting Trials (CONSORT) flow diagram of the study participants was used to show the process for selecting the participants which is given below (Figure 1).

The data presented in Table 1 reveals that the majority (60%) of the mothers were in the age group of 21-29, with a mean standard deviation of 21.80±3.327. Regarding socioeconomic class among these groups, a higher percentage belongs to the lower class. Almost all the participants live in joint families, and a higher percentage of participants from the intervention group live in the urban area of Bharatpur compared to the control group.

**Table 1:** Demographic information of the participants

Demographic information	Control group (n, %)	Intervention group (n, %)
Age (in years)		
18-20	2 (40)	2 (40)
21-29	3 (60)	3 (60)
Religion		
Hinduism	3 (60)	3 (60)
Buddhism	1 (20)	1 (20)
Other	1 (20)	1 (20)
Socioeconomic class		
Upper class	-	1 (20)
Lower middle class	1 (20)	2 (40)
Lower class	4 (80)	2 (40)
Family system		
Joint family	5 (100)	5 (100)
Residence		
Urban	2 (40)	4 (80)
Rural	3 (60)	1 (20)

The data presented in Table 2 represents the obstetrics information of the participants. Most of the participants in both groups have less than 18.5 BMI, i.e., 80%, before pregnancy. All the participants visited more than four times antenatal clinics for their checkups. Almost all participants were admitted to the hospital during their 39-40 weeks of gestation for delivery. Most of the participants (70%) had a latent phase of more than five hours, and labor started spontaneously among most of them. Regarding the presence of caregivers during labor, almost half of them had mothers as caregivers (50%), whereas very few had husbands as a primary caregiver.

**Table 2:** Obstetrics information of the participants

Variables	Control group (n, %)	Intervention group (n, %)
Body mass index		
Less than 18.5	4 (80)	4 (80)
18.5-24.9	1 (20)	1 (20)
Antenatal clinic visitation		
More than 4 times	5 (100)	5 (100)
Weeks of gestation		
39-40 weeks	4 (80)	5 (100)
41 weeks	1 (20)	-
Latent phase hours		
Less than 5 hours	2 (40)	1 (20)
5 hours and above	3 (60)	4 (80)
Type of labor started		
Spontaneous labor	3 (60)	3 (60)
Induction of labor	2 (40)	2 (40)
Presence of caregiver		
Husband	1 (20)	-
Mother	2 (40)	3 (60)
Mother-in-law	1 (20)	-
Immediate family	1 (20)	2 (40)

Table 3 displays the means, standard deviation, and F-value of labor pain before and after the application of heat therapy among primigravid women. The results indicated a significant mean difference in labor pain across four conditions (F (3,12)=4.500; MES=0.66; p=0.025; η2=0.53) with a large effect size. The findings revealed that a higher level of labor pain was decreased after the intervention as evident in post-test 2 (M=6.20; SD=0.83), post-test 3 (M=5.60; SD=0.89), and post-test 4 (M=5.00; SD=0.70). However, the pairwise comparison indicated that there are significant mean differences in pairs of scores between post-test 4.

**Table 3:** Mean, standard deviation, and repeated measures ANOVA for labor pain during the first stage of labor **: p<0.05; *: p>0.05 F (3,12): F-test or F-statistic which is a ratio of two variances in ANOVA and is needed to estimate the degree of freedom; η2: eta-squared needed to show the effect size in repeated measures ANOVA

Variables	Pre-test 1		Post-test 2		Post-test 3		Post-test 4		F (3,12)	P-value	η2
	M	SD	M	SD	M	SD	M	SD			
Intervention group	6.80	1.30	6.20	0.83	5.60	0.89	5.00	0.70	4.500**	0.025	0.529
Control group	7.00	1.22	7.20	0.44	7.00	0.70	6.80	0.44	0.242*	0.865	0.057

In contrast, the control group, who had received only standard care, showed no significant mean difference in labor pain across four conditions: F (3,12)=0.242, MES=0.42, p=0.865, and η2=0.057. The findings indicated that the labor pain levels during labor progression were not significantly reduced, as indicated by post-test 2 (M=7.20; SD=0.44), post-test 3 (M=7.00; SD=0.70), and post-test 4 (M=6.80; SD=0.44). However, the pairwise comparison indicated that there are no significant mean differences in all pairs of scores between tests 1, 2, 3, and 4.

**Figure 2:**
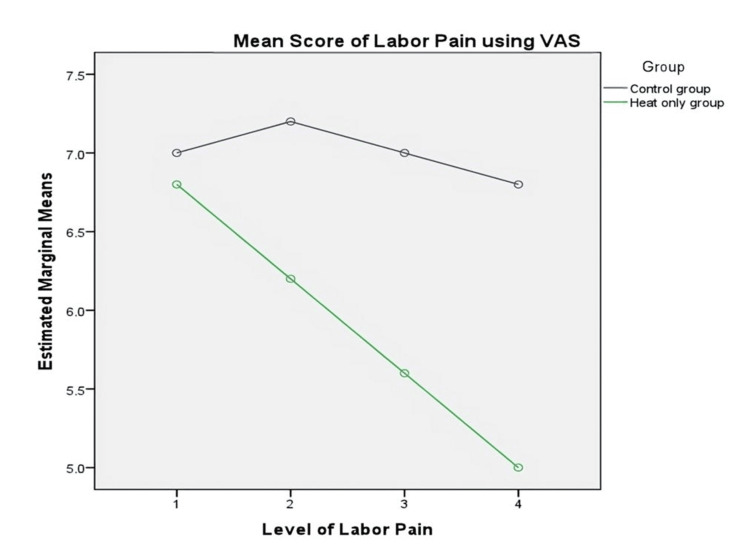
Comparison of the mean score of the level of pain during labor VAS: Visual Analog Scale

Figure 2 shows that the level of labor pain is significantly declining among the heat therapy group.

## Discussion

This pilot study, involving 10 low-risk primigravid women as participants, intended to compare the effectiveness of heat application on the level of pain throughout the active first phase of labor between the intervention and control groups. The mean and standard deviation of the participants' age were 21.80±3.327 which is relatively younger compared to a similar study conducted by Sayed et al. [13] in Egypt (25.76±1.76). This age difference may be attributed to the higher prevalence of early marriage in the country where the current study was conducted. The childbearing age and labor contribute to the labor pain-bearing capacity and experience among primigravid women.

BMI plays a significant role in labor for primigravid women. In the current study, most participants have a less-than-normal BMI (18.5-24.9) during the pre-pregnancy period, whereas, in a study conducted by Suthisuntornwong and Tangsiriwatthana [14] in Thailand, the maternal mean BMI of the intervention group was 28.1±3.3 and that of the control group was 26.9±3.7 which is higher than normal. The study by Tilden et al. [15] presented the opposing result that 66.6% of the intervention group had a normal BMI than that of the control group. Some research reported that labor complications are more common in those with higher BMI, which also raises exhaustion and labor pain perception. Similarly, the length of labor during the first stage also affects labor pain perception. The participants in the study experienced a latent phase lasting more than five hours for the intervention group (60%) and the control group (80%), which led to exhaustion among the laboring mothers. This current study supports the idea that a longer labor period increases fatigue [16].

The type of labor initiation has a significant effect on labor pain. In this study, 60% of both groups had spontaneous labor pain started. The presence of a caregiver during labor may help to deal with pain and discomfort. In this study, most of the primigravid women's caregivers were their own mothers.

The key purpose of the article was to compare the level of pain after heat application. The mean and standard deviation of pain in the intervention group were lower than those in the comparison group after the intervention, across three progressive labor stages, namely, post-test 2 (M=6.20; SD=0.83), post-test 3 (M=5.60; SD=0.89), and post-test 4 (M=5.00; SD=0.70), when assessed using the VAS. The findings were supported by various studies performed in different parts of the world. The mean labor pain score after an hour of intervention (6.77±128) in Turkey was much lower than that of the control group (8.57±1.01) [17]. In Taiwan, the labor pain score at 7 cm of cervical dilation was also lower than the control group (7.10±1.92 versus 8.85±1.22; p<.001) [18]. In Iran, labor experience was similar to the previous studies [19]. The study in Japan also supported the current study findings [20]. Similarly, the study in India [21] and Spain reported lower pain scores in the experimental group than in the control group [22]. Thus, this means heat therapy during labor helps to reduce labor pain perception. In our study, there is no significant difference at the first, second, and third assessments; however, there is a substantial difference at the fourth assessment which means that heat application during labor reduces pain at 9-10 cm of cervical dilatation, although other studies have a significant difference in pain perception after warm or heat application on the lower back [23,24].

Limitations of the study

This pilot study has a small sample size, which may limit the findings. Blinding was not feasible due to the nature of the study. Random allocation was used to control for variations in individuals' pain tolerance thresholds, which influenced how they responded to the pain level.

## Conclusions

This study has demonstrated that applying heat therapy to the lumbosacral area during the active first stage of labor effectively alleviated labor pain in primigravid women. After applying heat therapy, pregnant women's average pain scores significantly decreased as labor progressed, particularly in post-test 4, when compared to the control group. Heat therapy is, therefore, a useful choice for pain management to lessen the discomfort associated with labor, particularly for primigravid women.
